# Retraction: BRCA1 Regulates IFI16 Mediated Nuclear Innate Sensing of Herpes Viral DNA and Subsequent Induction of the Innate Inflammasome and Interferon-β Responses

**DOI:** 10.1371/journal.ppat.1010904

**Published:** 2022-10-19

**Authors:** The PLOS Pathogens Editors

Following the publication of this article [1], concerns were raised regarding results presented in multiple figures. Specifically,

- In Fig 3A, there appear to be vertical discontinuities suggestive of splice lines between lanes 4 and 5 in the TBP and Tubulin panels when colour levels are adjusted.
- In Fig 1Gb, the bands in lanes 1–4 of the IFI16 blot appear more similar to each other than would be expected from independent results.
- In Fig 4H, similarities are observed between lanes 2 and 3 of the Procaspase-1/Caspase-1 panel in regions beneath the Procaspase-1 bands.
- In Fig 14C (upper panel), there are similarities between data in the Si Cont and Si BRCA1 lanes for EdU labelled HSV-1.

The corresponding author provided files described as raw image data underlying some of the figures, and they indicated that not all the raw image data remain available.

The corresponding author also confirmed that the Fig 3A results presented in lanes 1–4 and lanes 5–6 were obtained from separate gels, and that the panel presents data spliced from separate images. The underlying data supporting the Fig 3A results are no longer available. The issue remains unresolved; however, the Editors are not concerned about the integrity of the data presented in this figure.

The corresponding author agreed that the regions of concern within Figs 1Gb, 4H, and 14C appear similar, and requested that the article be retracted.

In light of the concerns affecting multiple figure panels, the *PLOS Pathogens* Editors retract this article.

AR and BC agreed with the retraction, stand by the article’s findings, and apologize for the issues with the published article. All other authors either did not respond directly or could not be reached.
